# Deep learning approaches to predict 10-2 visual field from wide-field swept-source optical coherence tomography en face images in glaucoma

**DOI:** 10.1038/s41598-022-25660-x

**Published:** 2022-12-05

**Authors:** Sangwoo Moon, Jae Hyeok Lee, Hyunju Choi, Sun Yeop Lee, Jiwoong Lee

**Affiliations:** 1 Department of Ophthalmology, Pusan National University College of Medicine, Busan, 49241 Korea; 2 Biomedical Research Institute, Pusan National University Hospital, Busan, 49241 Korea; 3 Department of Medical AI, Deepnoid Inc, Seoul, 08376 Korea

**Keywords:** Optic nerve diseases, Retinal diseases, Vision disorders, Tomography, Computational science

## Abstract

Close monitoring of central visual field (VF) defects with 10-2 VF helps prevent blindness in glaucoma. We aimed to develop a deep learning model to predict 10-2 VF from wide-field swept-source optical coherence tomography (SS-OCT) images. Macular ganglion cell/inner plexiform layer thickness maps with either wide-field en face images (en face model) or retinal nerve fiber layer thickness maps (RNFLT model) were extracted, combined, and preprocessed. Inception-ResNet-V2 was trained to predict 10-2 VF from combined images. Estimation performance was evaluated using mean absolute error (MAE) between actual and predicted threshold values, and the two models were compared with different input data. The training dataset comprised paired 10-2 VF and SS-OCT images of 3,025 eyes of 1,612 participants and the test dataset of 337 eyes of 186 participants. Global prediction errors (MAE_point-wise_) were 3.10 and 3.17 dB for the en face and RNFLT models, respectively. The en face model performed better than the RNFLT model in superonasal and inferonasal sectors (*P* = 0.011 and *P* = 0.030). Prediction errors were smaller in the inferior versus superior hemifields for both models. The deep learning model effectively predicted 10-2 VF from wide-field SS-OCT images and might help clinicians efficiently individualize the frequency of 10-2 VF in clinical practice.

## Introduction

Glaucoma is a chronic progressive disease characterized by the progressive loss of retinal ganglion cells and their axons associated with structural changes of the optic nerve head and macula, which result in functional impairment of the visual field (VF)^1^.

In particular, VF defects within the central 10° area have a substantial impact on vision-related quality of life including facial recognition, locating objects, motion detection, and reading street signs^2,3^. However, 16% of eyes with normal 24-2 VF are classified as abnormal on 10-2 VF^4^. Therefore, close monitoring of central VF defects with 10-2 VF testing is important to prevent vision loss in patients with glaucoma.

Recently, computer technology has been markedly improved, and massive parallel computing capabilities have made it possible to handle deep learning models^5,6^. Several studies have predicted 24-2 VF from spectral domain (SD) or swept-source (SS) optical coherence tomography (OCT) using deep learning models^7–11^. Cristopher et al.^10^ reported that a deep learning model based on a retinal nerve fiber layer (RNFL) en face image outperformed other deep learning models in identifying glaucomatous 24-2 VF defects. Shin et al.^7^ found that a deep learning model estimated 24-2 VF better from an image of wide-field SS-OCT than from that of SD-OCT. Only two studies have predicted 10-2 VF from SD-OCT measurements using a deep learning model^12,13^.

However, previous studies used macular thickness measurements to predict 10-2 VF with a deep learning model^12,13^. Since the wider area measured by SS-OCT contains more information than the area measured by SD-OCT, the sensitivity and specificity were excellent in glaucoma diagnosis by better reflecting the structural damage corresponding to functional loss^7,14^. In addition, the voxel intensity values of the en face image contained information that could not be obtained from the thickness map, which measures only thickness^10^.

The purpose of this study was to develop a deep learning model to predict 10-2 VF from wide-field SS-OCT images and evaluate its performance.

## Methods

### Ethics

This study was approved by the institutional review board (IRB) of Pusan National University Hospital, South Korea (approval no. 2203-024-113) and registered at Clinical Research Information Service (approval no. KCT0007192). The requirement for patient consent was waived by the IRB of Pusan National University Hospital because of the retrospective nature of the study. This study was conducted in accordance with the tenets of the Declaration of Helsinki.

### Study design and population

All training and test dataset were obtained from individuals visiting the glaucoma clinic at Pusan National University Hospital from September 2015 to April 2021. The training dataset comprised 3025 eyes of 1612 participants. A separate, non-overlapping test dataset was prepared with 337 eyes of 186 participants. The demographic characteristics of the training and test groups are summarized in Tables 1 and 2.

**Table 1:** Demographics and clinical characteristics of the training group.

Number of eyes (patients)	3025 (1612)
Age, years	57.28 ± 15.26
Sex, Female	840 (52.11)
Best corrected visual acuity (logMAR)	0.15 ± 0.19
Intraocular pressure at test, mmHg	16.24 ± 4.37
Spherical equivalent, diopter	− 1.78 ± 3.15
Axial length, mm	24.42 ± 1.68
Central corneal thickness, μm	543.00 ± 40.55
Lens status, Phakia	2392 (79.07)
Diabetes mellitus	234 (14.52)
Hypertension	452 (28.04)
**Diagnosis**	
Normal	95
Glaucoma suspect	548
Ocular hypertension	169
Primary open-angle glaucoma	1640
Primary angle-closure glaucoma	216
Pseudoexfoliation glaucoma	164
Other secondary glaucoma	193
**10-2 visual field**	
MD, dB	− 5.08 ± 6.35
PSD, dB	4.32 ± 4.55
**SS-OCT**	
OCT image quality value	58.48 ± 5.86
**mGC/IPLT, μm**	
Average	61.88 ± 9.12
Superotemporal	62.15 ± 10.37
Superior	62.40 ± 9.65
Superonasal	67.52 ± 10.08
Inferonasal	63.83 ± 10.22
Inferior	56.53 ± 9.64
Inferotemporal	58.82 ± 12.25
**cpRNFLT, μm**	
Average	82.53 ± 21.13
Temporal	72.18 ± 18.72
Superior	99.16 ± 30.39
Nasal	63.38 ± 17.15
Inferior	95.37 ± 35.12

Values are presented as mean ± standard deviation (range) or number (%) unless otherwise indicated. *logMAR*  logarithm of the minimum angle of resolution; *MD*  mean deviation; *PSD*  pattern standard deviation; *SS-OCT*  swept-source optical coherence tomography; *mGC/IPLT*  macular ganglion cell/inner plexiform layer thickness; *cpRNFLT*  circumpapillary retinal nerve fiber layer thickness.

**Table 2:** Demographics and clinical characteristics of the test group.

Number of eyes (patients)	337 (186)
Age, years	63.69 ± 12.89
Sex, Female	103 (55.38)
Best corrected visual acuity (logMAR)	0.15 ± 0.25
Intraocular pressure at test, mmHg	16.32 ± 3.82
Spherical equivalent, diopter	− 1.23 ± 2.57
Axial length, mm	24.22 ± 1.52
Central corneal thickness, μm	543.23 ± 39.15
Lens status, Phakia	245 (72.70)
Diabetes mellitus	29 (15.59)
Hypertension	64 (34.41)
**Diagnosis**	
Normal	8
Glaucoma suspect	72
Ocular hypertension	15
Primary open-angle glaucoma	163
Primary angle-closure glaucoma	36
Pseudoexfoliation glaucoma	23
Other secondary glaucoma	20
**10-2 visual field**	
MD, dB	− 4.95 ± 6.23
PSD, dB	4.05 ± 4.28
**SS-OCT**	
OCT image quality value	57.19 ± 6.88
**mGC/IPLT, μm**	
Average	62.20 ± 9.45
Superotemporal	62.59 ± 10.66
Superior	62.59 ± 9.78
Superonasal	67.52 ± 10.07
Inferonasal	63.54 ± 10.66
Inferior	56.89 ± 9.70
Inferotemporal	60.05 ± 12.34
**cpRNFLT, μm**	
Average	83.31 ± 21.72
Temporal	71.61 ± 17.88
Superior	99.11 ± 29.67
Nasal	65.14 ± 18.74
Inferior	97.37 ± 36.27

Values are presented as mean ± standard deviation (range) or number (%) unless otherwise indicated. *logMAR*  logarithm of the minimum angle of resolution; *MD*  mean deviation; *PSD*  pattern standard deviation; *SS-OCT*  swept-source optical coherence tomography; *mGC/IPLT*  macular ganglion cell/inner plexiform layer thickness; *cpRNFLT*  circumpapillary retinal nerve fiber layer thickness.

For all participants, we retrospectively reviewed the detailed results of ophthalmic examinations, including diagnosis, age, sex, best corrected visual acuity (BCVA), intraocular pressure measurement with Goldmann applanation tonometry, keratometry with an Auto Kerato-Refractometer (ARK-510A; NIDEK, Hiroshi, Japan), central corneal thickness (Pachmate; DGH Technology, Exton, PA, USA), axial length (IOL Master, Carl Zeiss Meditec, Dublin, CA, USA), lens status, presence of diabetes mellitus, and presence of hypertension.

Glaucomatous optic neuropathy was defined if one or more of the following criteria were met: focal or diffuse neuroretinal rim thinning, localized notching, cup-to-disc ratio asymmetry ≥ 0.2, and presence of RNFL defects congruent with VF defects^15^. Normal participants were defined as those with no history of ocular disease, intraocular pressure < 21 mmHg, absence of a glaucomatous optic disc appearance, and normal VF.

Participants with retinal disease, neurologic disease, or severe media opacity that could affect VF and OCT measurement, such as diabetic retinopathy, age-related macular degeneration, corneal opacity, cataract, or refractive error ≥  ± 6.0 diopters, were excluded.

### SS-OCT

Wide-angle scanning using a deep range imaging (DRI) Triton SS-OCT device (Topcon, Tokyo, Japan) was performed on all participant within 6 months of 10-2 VF examination. Wide-field scanning involved the use of a wide-field 12- × 9-mm lens, with the scan centered on the fovea, for 256 B-scans, each comprising 512 A-scans, for a total of 131,072 axial scans per volume. Scan time of 1.3 s per 12- × 9-mm^2^ scan was used^5^. Poor-quality images (image quality scores < 40, poorly focused, or decentered during fovea scanning) or those acquired after segmentation failures or with artifacts owing to eye movements or blinking were excluded^5^.

### 10-2 VF test

Standard automated perimetry was performed on all participants using a Humphrey Visual Field Analyzer 750i instrument (Carl Zeiss Meditec) with the Swedish interactive threshold algorithm (SITA) standard 10-2. The 68 test points of the threshold value (THV) were used as the ground-truth VF of the training and test datasets. Reliable VF tests were defined as follows: false-positive rate < 20%, false-negative rate < 20%, and fixation loss < 33%^16^.

### Flow of the deep learning model

**Figure 1:**
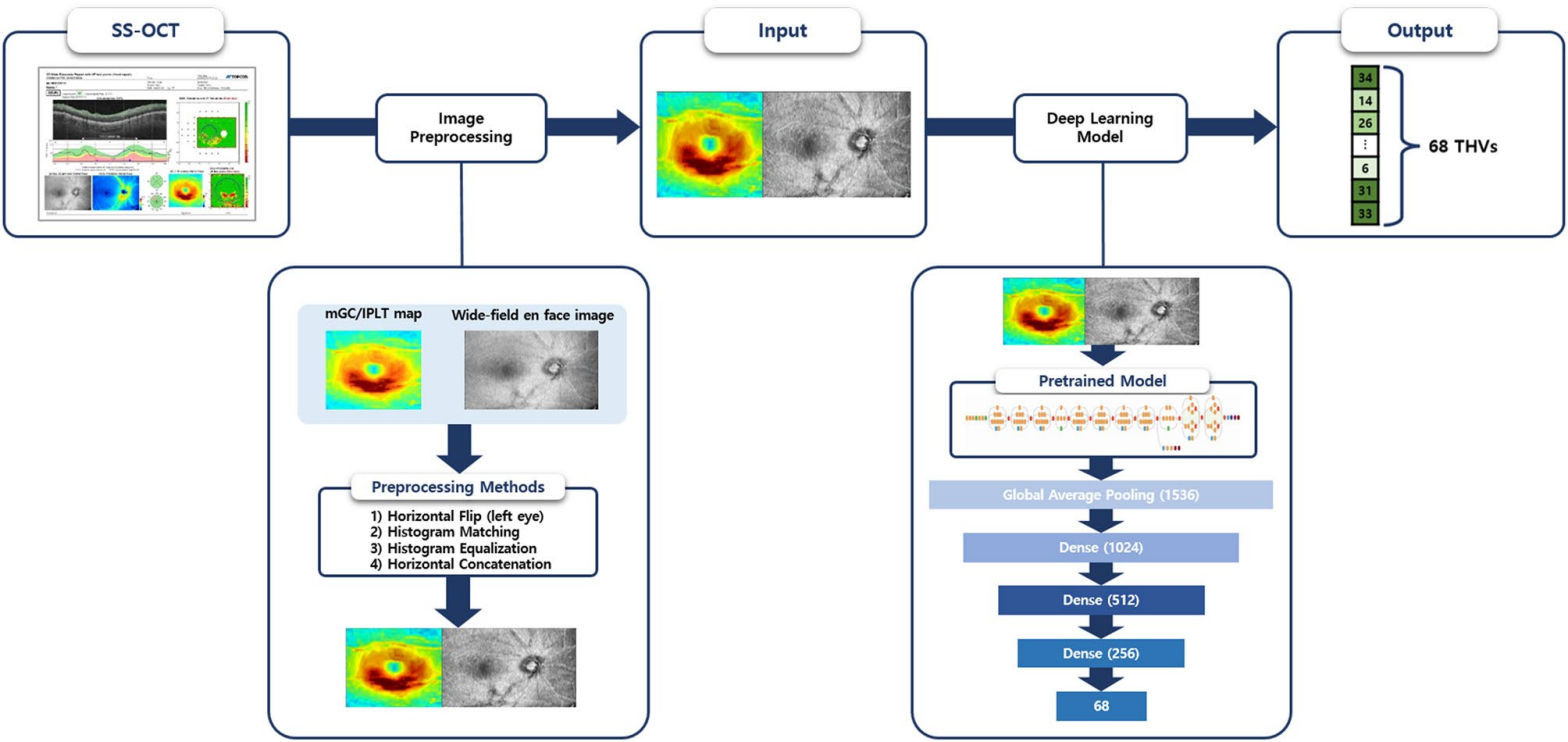
Flow diagram of the deep learning model (Google’s convolutional neural network architecture). Extraction of input images (wide-field en face image with mGC/IPLT map or wide-field RNFLT map with mGC/IPLT map) was performed on a wide-field scan of SS-OCT. Then, preprocessing of the extracted images was carried out, including consistency and contrast enhancement and concatenation of images. Inception-ResNet-V2 was used as the backbone structure at the beginning of the architecture to extract the global features. The global average pooling layer flattened the output of the Inception-ResNet-V2 backbone and averaged 1536 features. The three dense layers gradually condensed these features into 68 final output values, which corresponded to 68 10-2 visual field threshold values. *mGC/IPLT*  macular ganglion cell/inner plexiform layer thickness; *RNFLT*  retinal nerve fiber layer thickness; *SS-OCT*  swept-source optical coherence tomography.

The deep learning model to predict 68 THVs of 10-2 VF from wide-field SS-OCT images comprised a three-step process: (1) extraction of input images from a wide-field SS-OCT scan, (2) preprocessing of the extracted images, including enhancement of consistency and contrast, and concatenation of images, and (3) prediction of THVs using a deep learning model (Fig. 1).

### Input image generation

We developed an algorithm to extract images automatically using the Pillow module, an image processing library of Python, to be used as input data for the deep learning model. Our algorithm used three exported SS-OCT images: (1) macular ganglion cell/inner plexiform layer thickness map (mGC/IPLT map) 200 × 200 (width, height), (2) wide-field en face image 280 × 200, and (3) wide-field RNFL thickness (RNFLT) map 280 × 200. All left eye images were flipped horizontally to match the right eye format.

Preprocessing was performed so that the deep learning model could more efficiently predict 10-2 VF from the extracted images. Techniques to improve image consistency and contrast between images were implemented, and two different images were then concatenated. The final combined image had a resolution of 480 × 200 pixels (Supplementary Fig. S1).

Differences in image brightness and contrast could potentially affect deep learning model performance because the model performs prediction through a matrix calculation for each pixel of the image. To address this, a histogram-matching technique was performed to match the pixel probability distribution of the images representing brightness and contrast between images^17^.

On the mGC/IPLT map, wide-field RNFLT map (showing retinal thickness as a heat map), and wide-field en face image (expressing difference in brightness), the contrast of pixel values is an important factor to determine the degree of VF damage. Therefore, contrast limited adaptive histogram equalization (CLAHE) was used as enhancement^17,18^.

A wide-field en face or wide-field RNFLT image was combined with an mGC/IPLT image to form one input image. The combined image, sized 480 × 200, was fed into the input layer of the deep learning model.

### Deep learning model and training

The deep neural network architecture predicting the 10-2 VF THV through preprocessed input data (horizontally concatenated image) is shown in Fig. 1. The open source deep learning library, Ubuntu Linux-based operating system (version 18.04.5 LTS) and Python (version 3.8.10, Python Software Foundation) was used.

A convolutional neural network architecture, Inception-ResNet-V2, was used as the backbone. Before training, the pretrained ImageNet weight of the Inception-ResNet-V2 was downloaded and applied. A bottleneck layer of the backbone was modified by one global average pooling layer followed by three consecutive fully connected layers (dense layers 1–3 in Fig. 1). The rectified linear unit (ReLu) was used as the activation function in all three dense layers. In this study, the parameters of both the backbone model and the fully connected layer were fine-tuned. Since the images of 'ImageNet' were used to set the initial value of weights in the pre-trained model and have different characteristics and purposes from the SS-OCT image, the weights of the convolutional layer were set to be trainable without freezing. Through a preliminary experiment, it was confirmed that the fine-tuning improved performance compared to the case of freezing.

The size of the output data for each layer is presented in Supplementary Fig. S2. As the input image passes through the backbone architecture (Inception-ResNet-V2), a shape of (13,4,1536) was created, and a one-dimensional layer with the length of the number of channels in the last convolutional layer was formed through global average pooling. After that, the output was produced through 3 fully connected layers. Three dense layers gradually condensed these features into 68 final output values (THV)^11^.

The 3362 records from the entire dataset were randomly split into training, validation, and test groups in a 8:1:1 ratio. Validation dataset were used to check the current fitness of the neural network during training to prevent overfitting. For the training model, 300 epochs with a batch size of 16 were supplied to the neural network. For the loss function, mean squared error was used. When no further performance gain was observed over 300 epochs, training was completed. The optimizer was ‘RMSProp’ and learning rate was set to 0.001. The learning rate decay was set to 0.9 every 20 epochs; this identified the optimal minimum point by lowering the learning rate as learning time increased. For training process monitoring, the loss and mean absolute error (MAE) values for each training and validation dataset were confirmed at every epoch.

The deep learning model extracted the output through 3 fully connected layers after the backbone model (Inception-ResNet-V2) and global average pooling (Fig. 1) (Supplementary Fig. S2)^19^. Weights were then calculated through matrix multiplication of these 3 fully connected layers. Based on the weighted sum of the last convolutional layers of the backbone model, a heat map about one specific class image was generated^19^. With this Class Activation Mapping (CAM) technique, we confirmed which area of the input image the deep learning model focused on to predict each of the 68 THVs of the 10-2 VF.

### Statistical analysis

**Figure 2:**
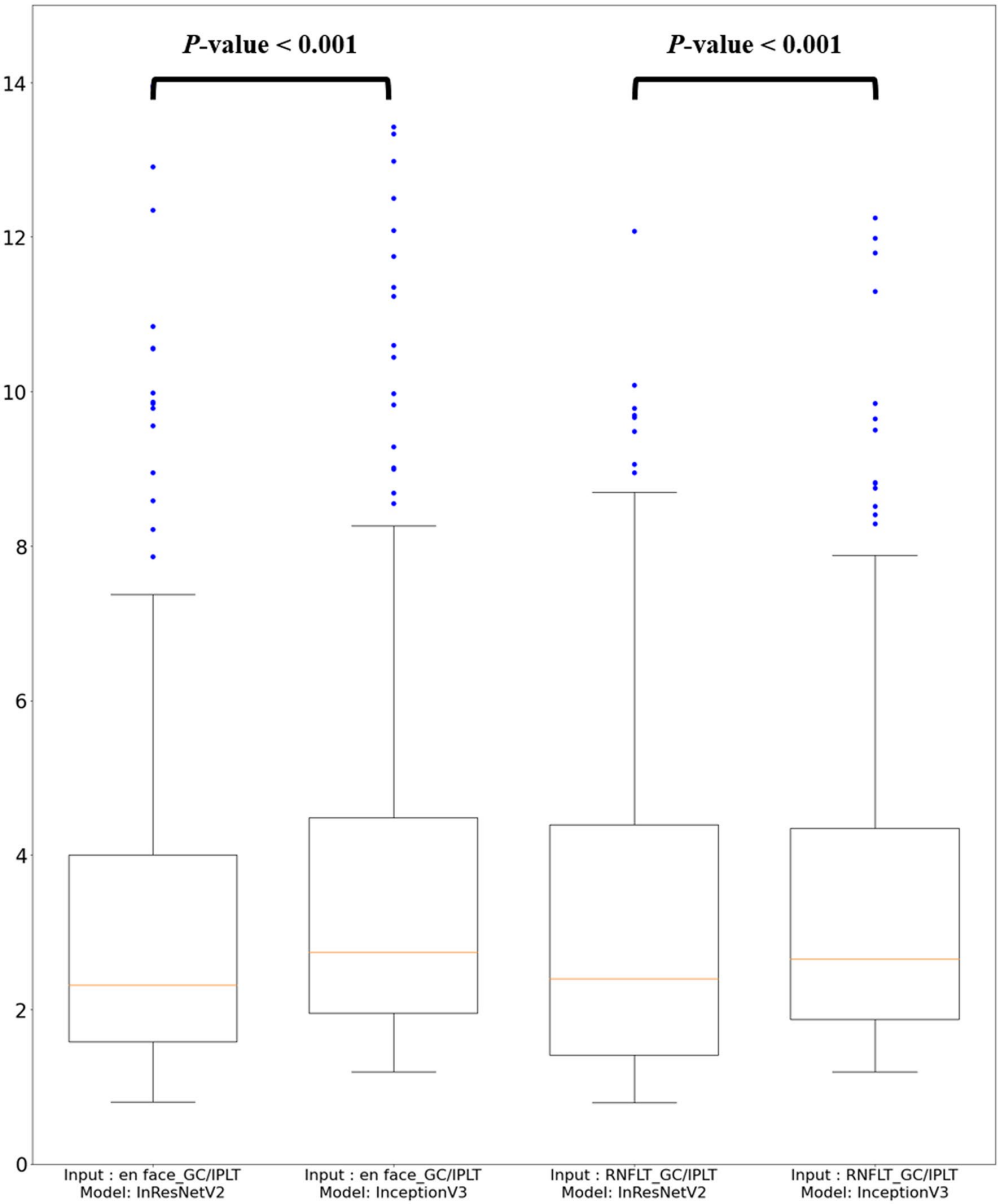
Global mean absolute error (MAE_point-wise_) of 10-2 visual field prediction from Inception-ResNet-V2 and Inception V3. The prediction errors of Inception-ResNet-V2 were significantly lower than those of Inception V3 regardless of input images. *mGC/IPLT*  macular ganglion cell/inner plexiform layer thickness; *RNLFT*  retinal nerve fiber layer thickness; *InResNetV2*  Inception-ResNet-V2.

The Shapiro–Wilk test was performed to check data distribution normality. To compare performance between the en face model (a wide-field en face image with mGC/IPLT map) and RNFLT model (a wide-field RNFLT map with mGC/IPLT map), we performed the paired t-test or Wilcoxon’s signed-rank test depending on data normality. To compare prediction performance, MAE values of the THVs were used as accuracy metrics. On preliminary analysis, Inception-ResNet-V2 had a lower global prediction error (MAE) than Inception V3 (Fig. 2). Therefore, in this study, Inception-ResNet-V2 was used as the backbone model and trained to predict 10-2 VF from combined images.

### Global analysis

Absolute error (AE) between predicted and actual THV was calculated for the 68 test points, and the mean of the 68 AEs was calculated. Global MAE based on point-wise analysis (MAE_point-wise_) was calculated per eye using the following Eq. (1):

1 $$ {\text{MAE}} = \;{ }\frac{1}{68}\mathop \sum \limits_{{{\text{n}} = 1}}^{68} \left| {{\text{true}}\;{\text{THV}}_{{\text{n}}} \; - \;{\text{predicted}}\;{\text{THV}}_{{\text{n}}} } \right| $$

$$ {\text{n}}\; = \;{\text{n}}^{{{\text{th}}}} { }\;{\text{point }}\;{\text{of }}\;{\text{visual }}\;{\text{field }}\;{\text{exam}} $$

$$ {\text{THV}}\; = \;{\text{visual }}\;{\text{field }}\;{\text{threshold }}\;{\text{value}} $$

### Sectoral analysis

**Figure 3:**
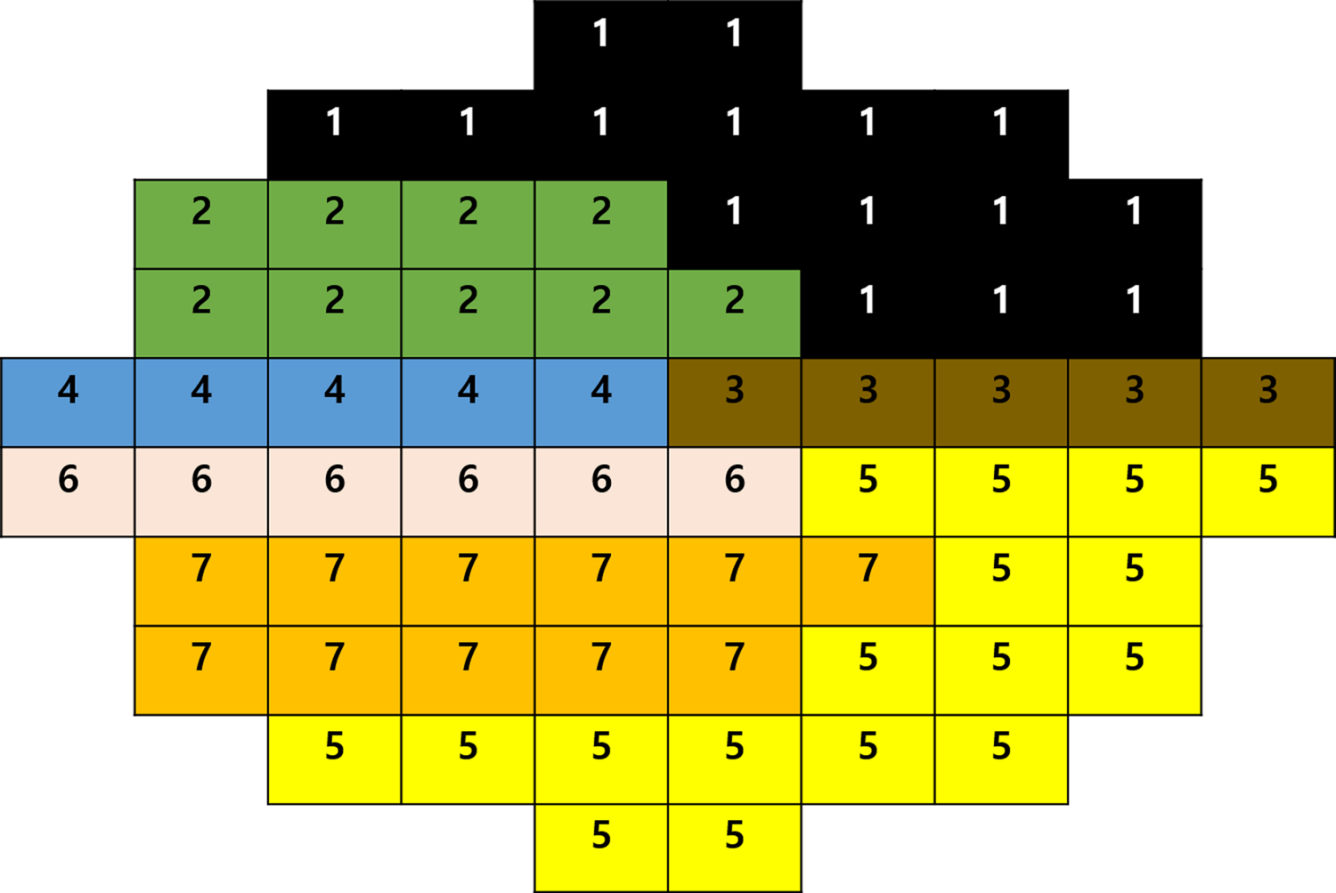
Cluster map of 10-2 visual field for sectoral analysis.

Sixty-eight test points were clustered into seven sectors using the cluster map proposed by de Moraes et al.^20^ (Fig. 3). To obtain the mean threshold sensitivities in each sector, threshold sensitivity in dB units at each of the 68 VF locations was first converted to a linear scale (1/Lambert) with the following formula: QUOTE . The values of all test points within each sector were averaged per eye. Then, the average VF sensitivity per sector was converted back to the dB scale for analysis. Sectoral analysis used the same deep learning model as global analysis, and training and evaluation were conducted as new models. In addition, global MAE based on sectoral analysis (MAE_sector_) was calculated per eye using the following Eq. (2):

2 $$ {\text{MAE}} = { }\frac{1}{7}\mathop \sum \limits_{{{\text{n}} = 1}}^{7} \left| {{\text{true}}\;{\text{THV}}_{{\text{n}}} \; - \;{\text{predicted}}\;{\text{THV}}_{{\text{n}}} } \right| $$

$$ {\text{n}} = \;{\text{n}}^{{{\text{th}}}} { }\;{\text{sector }}\;{\text{of }}\;{\text{visual }}\;{\text{field }}\;{\text{exam}} $$

$$ {\text{THV}}\; = \;{\text{visual }}\;{\text{field }}\;{\text{threshold }}\;{\text{value}} $$

### Location-wise analysis

MAE was calculated per VF test point of all test dataset eyes according to input data for comparing prediction performance using the following Eq. (3):

3 $$ MAE_{n} = \mathop \sum \limits_{i = 1}^{number\; of \;eyes} \frac{{\left| {true\; THV_{i, n} \; - \;predicted\; THV_{i, n} } \right|}}{number \;of\; eyes} $$

$$ n\; = \;n^{th} \;test \;point \;of \;visual \;field\; exam, \;i = {\text{i}}^{{{\text{th}}}} \; eye $$

$$ THV_{i, n} \; = \;threshold\; value \;of\; i^{th} \; eye,\; n^{th} \; test \;point $$

In all statistical analyses, SPSS (version 22.0 for Windows; SPSS, Chicago, IL, USA) was used, and a *P* value of < 0.05 indicated statistical significance.

## Results

Global and sectoral VF estimation error between ground truth and estimation according to two different input images (wide-field en face image with mGC/IPLT map or wide-field RNFLT map with mGC/IPLT map) are summarized in Table 3. Global MAE_point-wise_ was 3.10 ± 2.40 dB (mean ± standard deviation) and 3.17 ± 2.37 dB for the en face and RNFLT model, respectively (*P* = 0.287). Global MAE_sector_ was 2.64 ± 2.36 dB and 2.68 ± 2.29 dB for the en face and RNFLT model, respectively (*P* = 0.757). Globally, the estimation errors did not differ significantly between the two models. On sectoral analysis, the prediction error of the en face model was significantly lower than that of the RNFLT model in sectors 4 and 7 (*P* = 0.011 and *P* = 0.030, respectively). The lowest MAE sector was sector 5 (inferotemporal area) in both en face and RNFLT models.

**Table 3:** Global and the sectoral mean absolute error of 10-2 visual field prediction according to input images using Inception-Resnet-V2.

	En face model	RNFLT model	*P* value
Global MAE_point-wise_	3.10 ± 2.40	3.17 ± 2.37	0.287
Global MAE_sector_	2.64 ± 2.36	2.68 ± 2.29	0.757
**Sector proposed by de Moraes et al**.^20^			
1	2.56 ± 2.87	2.51 ± 2.57	0.150
2	2.58 ± 3.04	2.42 ± 2.77	0.742
3	3.13 ± 3.91	2.96 ± 3.72	0.433
4	2.31 ± 2.81	2.44 ± 2.89	0.011*
5	1.96 ± 2.06	2.01 ± 1.97	0.358
6	2.12 ± 2.68	2.23 ± 2.61	0.135
7	2.07 ± 2.43	2.25 ± 2.61	0.030*

Values are presented as mean ± standard deviation. Significance is marked in * (*P* value < 0.05).

*MAE*  mean absolute error, *RNFLT*  retinal nerve fiber layer thickness.

**Figure 4:**
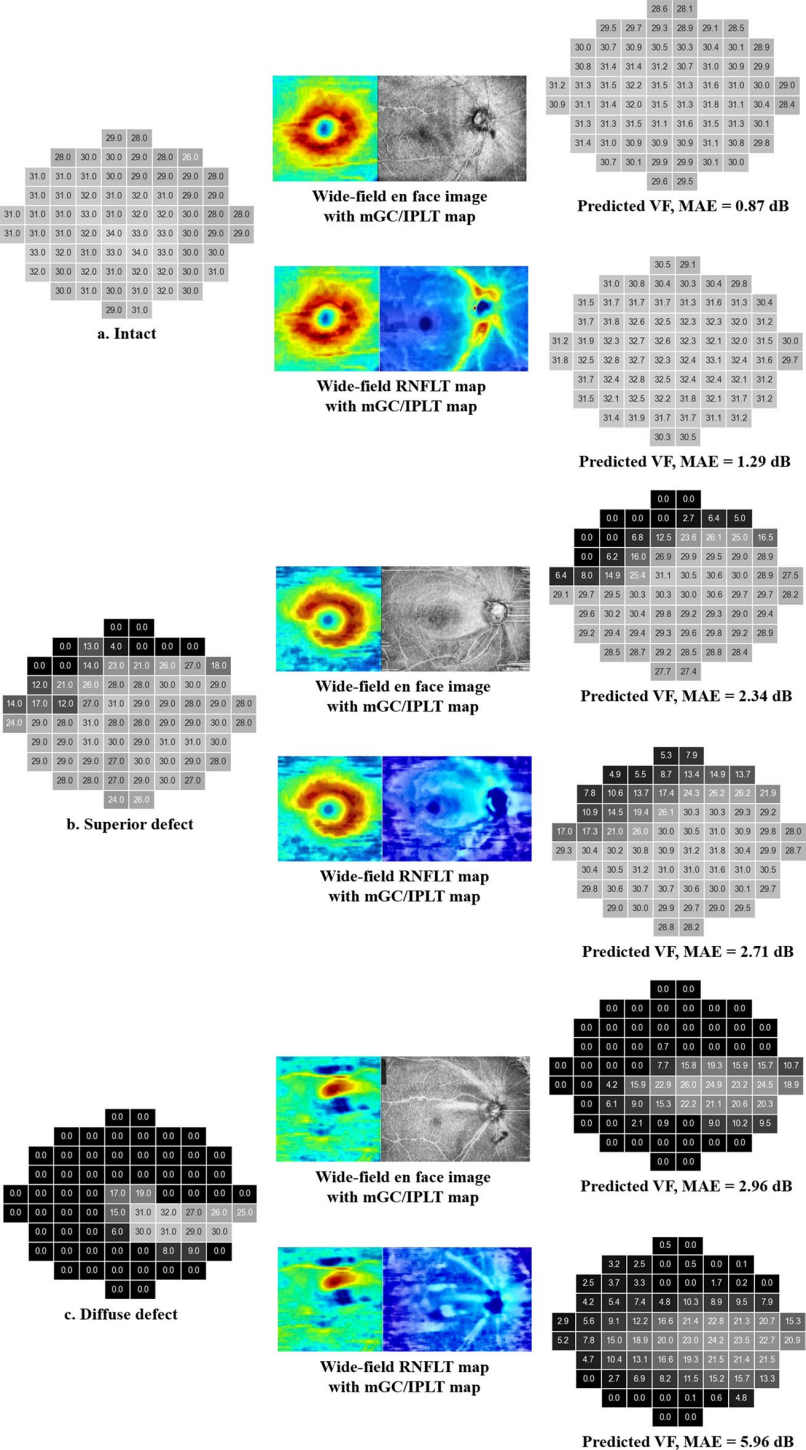
Representative cases of 10-2 visual field (VF) prediction. The actual threshold values (THVs) of 10-2 VF tests are represented in the left column (**a**, **b**, **c**). The combined OCT images are represented in the middle column. The THVs predicted by the en face model (upper) and RNFLT model (lower) are represented in the right column. Darker color represents lower THV (left and right columns). *mGC/IPLT*  macular ganglion cell/inner plexiform layer thickness; *RNLFT*  retinal nerve fiber layer thickness; *MAE*  mean absolute error.

According to location-wise analysis, the actual THVs were higher and prediction error lower in the inferior hemifield than in the superior hemifield for both models (Supplementary Fig. S3). Although the prediction errors did not differ significantly between the two models (all *P-values* ≥ 0.061, Wilcoxon’s signed-rank test), the en face model showed lower prediction errors at 44 test points in the superior VF that correspond to the more vulnerable zone proposed by Hood et al.^21^ (Supplementary Fig. S3d). Representative cases of 10-2 VF prediction are shown in Fig. 4. Although the deep learning models have never seen the actual 10-2 VF, the predicted 10-2 VF looked very similar to the actual VF.

**Figure 5:**
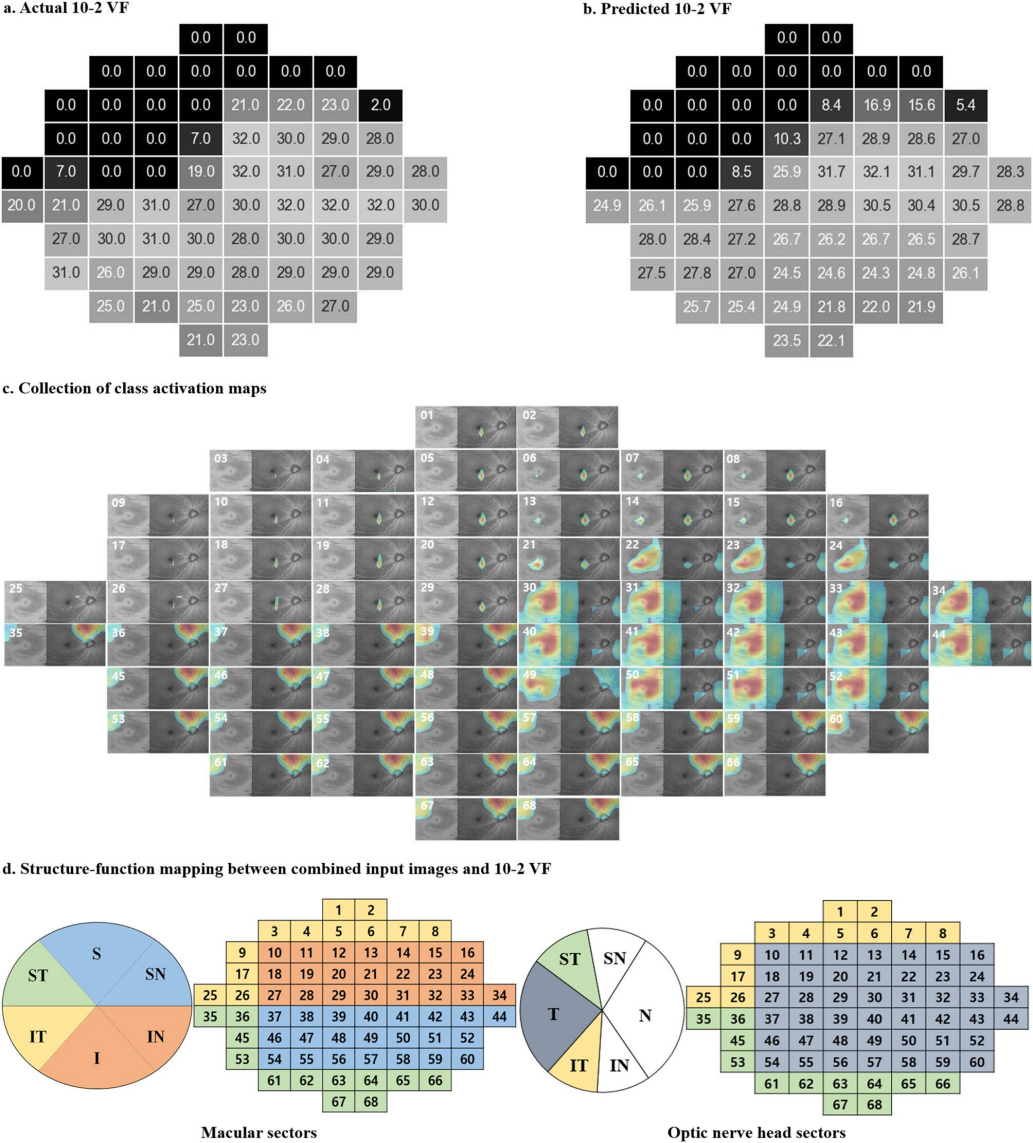
Representative example of Class Activation Mapping (CAM) with the en face model. Sixty-eight class activation maps were placed at the individual 10-2 visual field (VF) test points (right eye). The figure shows the actual 10-2 VF threshold values (THVs) and the predicted THVs (**a**, **b**). Each CAM image is numbered at the top left (**c**). The red color indicates the region where the deep learning model was highly activated and generated a high sensitivity value for the 10-2 VF test point, whereas the blue color indicates the opposite (**c**). Structure–function mapping between combined input images (including macula and optic nerve head scan; each in the left column) and 10-2 VF (right column) (**d**). The macula and optic nerve head sectors and the corresponding 10-2 VF regions are indicated with similar color^27^. The numbers in the VF images are the same as those in the CAM images.

The representative case of CAM is represented in Fig. 5. In each CAM image, red indicates strongly activated points yielding high THVs and blue (or no color) indicates the opposite. In this example, VF loss was predominantly found in the superonasal area of the actual and predicted VF (Fig. 5a,b, respectively). The majority of VF test points in superonasal area have THVs of zero and are expressed as black squares in the actual and predicted VF. The inferior and some superotemporal regions are relatively intact (i.e., exhibiting high THV). The CAM images numbered 21–24 and 30–68 were intensely red (Fig. 5c) and generate high THVs (Fig. 5b). Note that these activated areas in the CAM images exactly match the corresponding green, blue, and gray regions (Fig. 5d). In contrast, the CAM images numbered 1–20 and 25–29 were not colored (and thus not activated) (Fig. 5c) and generate no to low THVs (Fig. 5b). These areas match the corresponding yellow, orange, and gray regions (Fig. 5d).

Correlation analysis was performed to determine the factors affecting 10-2 VF prediction (Supplementary Table S1). The prediction error (global MAE_point-wise_) was positively correlated with BCVA and negatively correlated with the spherical equivalent, 10-2 VF mean deviation (MD), OCT image quality, average mGC/IPLT, and circumpapillary RNFLT (cpRNFLT) in both models. As glaucoma progressed, estimation performance was worse in both models. Supplementary Figure S4 shows the relationship between prediction error and 10-2 VF MD using scatter plots.

Multiple linear regression analyses were performed to investigate the relative influence of possible factors affecting 10-2 VF prediction (Supplementary Table S2). The models were constructed using the enter method and with global MAE_point-wise_ as the outcome variable. Age, BCVA, spherical equivalent, CCT, axial length, 10-2 VF MD, OCT image quality, average mGC/IPLT, and cpRNFLT were used as independent variables. No multicollinearity was found between the variables (all variance inflation factors ≤ 5.414). The 10-2 VF MD was the most influential variable in the en face (β =  − 0.701, *P* < 0.001) and the RNFLT models (β =  − 0.588, *P* < 0.001); it was followed by the average mGC/IPLT (β =  − 0.210, *P* = 0.048) in the RNFLT model.

## Discussion

Our results indicate that the deep learning model accurately predicted 10-2 VF from SS-OCT images. In point-wise analysis, prediction errors in the inferior hemifield were smaller than those in the superior hemifield for both models. However, the en face model showed better estimation performance than the RNFLT model in the superior hemifield that corresponds to the more vulnerable zone of the macula. Moreover, on sectoral analysis, the en face model showed better estimation performance than the RNFLT model in sectors 4 and 7.

Several studies have recently estimated 24-2 VF from SD-OCT or SS-OCT images using deep learning models^5,7,10,11^. Only two studies have estimated 10-2 VF from SD-OCT thickness measurements using a deep learning model^12,13^. However, the prediction of 10-2 VF using OCT image was not performed in the previous studies^5,7,10–13^. Hashimoto et al.^12^ utilized a deep learning method to estimate 10-2 VF from SD-OCT thickness measurements. The deep learning model performed better than machine learning or multiple linear regression models, with a global prediction error (MAE) of 5.47 dB. In the second study, Hashimoto et al.^13^ used actual THVs of 24-2/30-2 VF to correct the predicted THVs of 10-2 VF with a deep learning model and the global prediction error (MAE) after correction decreased to 4.2 dB.

We observed lower prediction errors with our deep learning model than those reported for the models by Hashimoto et al.^12,13^. The global prediction errors (MAE_point-wise_) were 3.10 dB (en face model) in this study, and 5.47 and 4.2 dB in theirs^12,13^. On sectoral analysis, all sectoral prediction errors were lower in our study than in a previous study^12^. On location-wise analysis, prediction errors were also lower at most of the 68 test points in the current versus previous studies^12,13^. Although our prediction errors within the superior hemifield, corresponding to the more vulnerable zone, were greater than those of inferior hemifield (the less vulnerable zone), they still remained lower than those of the preceding studies^12,13^.

There are several potential explanations of why the en face model of the present study achieved better results than the models of Hashimoto et al.^12,13^.

First, the scanning area of SS-OCT (12 × 9 mm including macula and optic disc) is wider than that of SD-OCT (9 × 9 mm centered on fovea). Shin et al.^7^ found that wide-field SS-OCT images were significantly more accurate than SD-OCT images at predicting a 24-2 VF. The authors suggested that wider scanning area of SS-OCT should contain much more information than the area of SD-OCT. Other studies have reported that the wide-field scan of SS-OCT collects the information needed to diagnose glaucoma with excellent sensitivity and specificity^14,22,23^. Therefore, a wide-field scan of SS-OCT images should better reflect the structural damage that corresponds to functional loss than the more narrow scan of SD-OCT.

In contrast to previous studies, which have used thickness measurements of the macular area alone^12,13^, we predicted 10-2 VF from SS-OCT images including both the macula and optic disc. Previous studies have found that mGC/IPLT correlated well not only with cpRNFLT but also the central VF, within 7.2° of the fovea^24,25^. Lee et al.^26^ have reported that 10-2 VF test points were mostly overlapped on a macular OCT scan (central 4.8 × 4.0 mm) and correlated with each other. Jung et al.^27^ have also shown structure–function correspondence maps between 10-2 VF test points and regions of the ONH or mGC/IPLT maps. Therefore, these two ONH and macular OCT scans may have complementary roles in predicting 10-2 VF.

We analyzed the performance the Inception-ResNet-V2 trained on mGC/IPLT map alone or en face image alone. And then we compared the performance of the models using single kind of image with that of the model using the combination of mGC/IPLT with en face image. We found that the model using the combination of mGC/IPLT with en face image significantly outperformed the model using either mGC/IPLT map alone or en face image alone. The global MAE_point-wise_ of the models using mGC/IPLT map alone (3.62 ± 2.99 dB) and en face image alone (3.22 ± 2.51 dB) were significantly greater than that of the model using combination of mGC/IPLT map with en face image (3.10 ± 2.40 dB) (*P* < 0.001 and *P* = 0.041, respectively). The global MAE_sector_ of the models using mGC/IPLT map alone (3.11 ± 2.86 dB) and en face image alone (2.79 ± 2.49 dB) were significantly greater than that of the model using combination of mGC/IPLT map with en face image (2.64 ± 2.36 dB) (*P* = 0.001 and* P* = 0.046, respectively).

Second, OCT images have additional information including RNFL reflectivity and the location of the major vessels associated with the RNFLT profile and bundle geometry that are not offered by thickness measurements alone^28,29^. Shin et al.^30^ observed that the diagnostic abilities of a deep learning classifier based on wide-field SS-OCT images outperformed that of a conventional parameter-based method. Lazaridis et al.^31^ reported that the deep learning model based on OCT images along with RNFLT measurements showed lower 24-2 VF prediction errors than a model based on RNFLT measurements alone.

The en face model outperformed the RNFLT model in sectoral analysis. The en face image may detect localized glaucomatous damage that could be missed or easily overlooked on the RNFLT map^14,32^. Christopher et al.^10^ suggested that an en face image has additional information provided by voxel intensity values within the RNFL, not available through the thickness map alone. Unlike RNFLT measurements, which depend solely on thickness, en face images may reflect abnormalities of both thickness and reflectance intensity^32^. A previous study demonstrated a saturation effect in the structure–function relationship of glaucoma due to residual glial cells and blood vessels that provide mGC/IPLT or RNFLT even after complete loss of visual function^33^. This remnant thickness may interfere with VF estimation from mGC/IPLT or RNFLT. In representative cases with VF defects (Fig. 4), despite some test points with complete loss of visual function (0 dB of THV), the RNFLT model showed various THVs. On the other hand, the en face model predicted THVs at those test points that were more similar to the actual values. In addition, automated segmentation errors have often been observed on the RNFLT map, and the proximal RNFL boundary with the ganglion cell layer is difficult to identify^32^. In comparison, the en face image was obtained by taking the average reflectance intensity of a 52-μm-thick slab below the internal limiting membrane, which was the easiest border for automated algorithms to identify reliably^32,34^.

Third, in this study, we used an image preprocessing process that allowed the deep learning model to more efficiently predict 10-2 VF. First, a histogram-matching technique was used to improve the issue of unbalanced pixel distribution between images. In the histogram-matching technique, given one representative pixel distribution, the algorithm identifies color mapping that optimally transforms another histogram into that one. Through this, all images are matched with one specific pixel distribution to achieve consistency^17^. Next, we used histogram equalization to evenly disperse the pixel distribution in a narrow spectrum over the entire range to enhance the contrast of the image. However, pixels are modified by a transform function based on the intensity distribution of the entire image. Thus, typical histogram equalization is suitable for enhancing the overall contrast but may cause issues in enhancing image details. To improve this, we used CLAHE, a preprocessing technique that divides the image into small blocks of a certain size and applies histogram equalization to each block^17,18^. In addition, previous studies reported that image preprocessing improved the performance of the deep learning model in detecting coronavirus infected pneumonia^35,36^.

We found that 10-2 VF prediction error became greater as glaucoma progressed. On regression analysis, 10-2 VF MD and average mGC/IPLT were negatively correlated to the global prediction error. This result is consistent with those of previous studies reporting that 24-2 VF MD was significantly associated with 24-2 VF prediction error^7,11^.

This study has some limitations. First, all 10-2 VF and SS-OCT data were acquired from Korean patients at a single center. Girkin et al.^37^ have reported that the measurements of ONH, RNFL, and macular parameters vary by race. Thus, our deep learning method may not be widely applicable to other ethnic groups. Future work on multi-central datasets would enable us to determine the generalizability of these deep learning models. Second, visual interpretability is always the Achilles’ heel of deep neural networks and ongoing area of research^38^. High model interpretability could help us provide detailed information about structure–function relationships. Our deep learning model generated a structure–function map by itself during the training process. In Fig. 5c, the CAM showed how a deep learning model constructed this map and indicated that the structure–function relationship was similar to that in previous studies^21,27^. Furthermore, the deep learning model considers not only specific mapping spots but also broad neighboring areas of OCT images. Finally, our study reported prediction errors for single SS-OCT/10-2 VF pairs. A larger proportion of the prediction error was attributable to the intrinsic variability of the 10-2 VF examination per se as well as to the prediction^7,31^.

In conclusion, the current study demonstrated that the deep learning models accurately predicted the 10-2 VF from the SS-OCT images. The results obtained here suggest that this deep learning model can mitigate the need for 10-2 VF examination by utilizing SS-OCT imaging. We believe that precisely predicting the central VF loss from SS-OCT imaging might help clinicians efficiently individualize the frequency of 10-2 VF testing to a single patient in clinical practice and, thus, contribute to efforts for preventing vision loss in glaucoma patients.

## Supplementary Information

Supplementary Information.

## Data Availability

The data generated or analyzed during this study are available from the corresponding author [J.L] upon reasonable request.
