# Supplementary material for: Deep learning approaches to predict 10-2 visual field from wide-field swept-source optical coherence tomography en face images in glaucoma
Source: Sci Rep. 2022 Dec 5;12:21041. doi: 10.1038/s41598-022-25660-x (PMC9722778; doi:10.1038/s41598-022-25660-x)
Supplement: Supplementary file 1 — Supplementary Information. [file 41598_2022_25660_MOESM1_ESM.docx]

**Deep learning approaches to predict 10-2 visual field from wide-field swept-source optical coherence tomography en face images in glaucoma**

**Sangwoo Moon, MD^1,2^, Jae Hyeok Lee^3^, Hyunju Choi^3^, Sun Yeop Lee^3^, and Jiwoong Lee, MD, PhD^1,2,^***

^1^Department of Ophthalmology, Pusan National University College of Medicine, Busan 49241, Korea

^2^Biomedical Research Institute, Pusan National University Hospital, Busan 49241, Korea

^3^Department of Medical AI, Deepnoid Inc, Seoul, 08376, Korea

*****[glaucoma@pnu.ac.kr](mailto:glaucoma@pnu.ac.kr)

Supplementary Fig. S1. Input image generation with extraction and preprocessing. After automatically extracting images (**a**), techniques to improve consistency and contrast between images were used, and two different images were concatenated (**b**). The final combined image has a resolution of 480 × 200 (width × height) pixels.

**a. Before preprocessing b. After preprocessing**


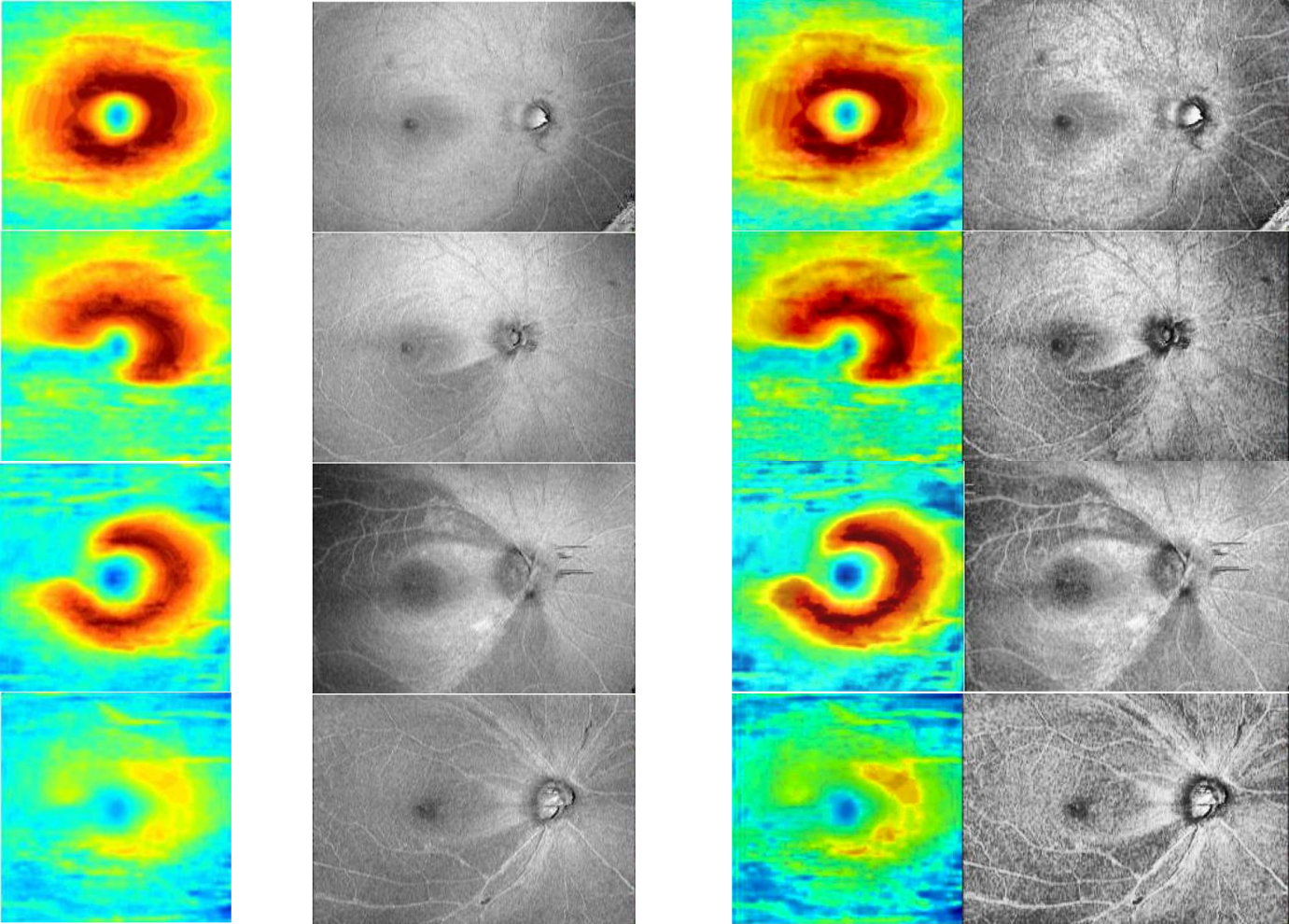


Supplementary Fig. S2. Size of each output feature through deep learning model. The output of the Inception-ResNet-V2 backbone architecture produced a shape of 13 × 4 × 1536 (width × height × depth) global features. The global average pooling layer flattened the backbone output and averaged 1536 features. Three dense layers gradually condensed these features into 68 final output values.

mGC/IPLT = macular ganglion cell/inner plexiform layer thickness; RNFLT = retinal nerve fiber layer thickness

Supplementary Fig. S3. Actual threshold values (THVs) and prediction error at each test point with the deep learning models. Actual THVs in central 10-2 visual field (right eye) (**a**). Location-wise absolute error of central 10-2 visual field prediction by deep learning model (en face and RNFLT model) (**b**, **c**). The difference in prediction errors between the two models: better results using an en face model marked in white gray scale and those using RNFLT model marked in dark gray scale (**d**). All left eyes were flipped horizontally. The darker the color is, the lower THVs (**a**) or the greater estimation error (**b**, **c**). Values are presented as mean THV (upper**; a**) or mean absolute error (upper; **b**, **c**) ± standard deviation (lower; **a**, **b**, **c**) at each location. RNFLT = retinal nerve fiber layer thickness

**a. Actual threshold values b. En face model**


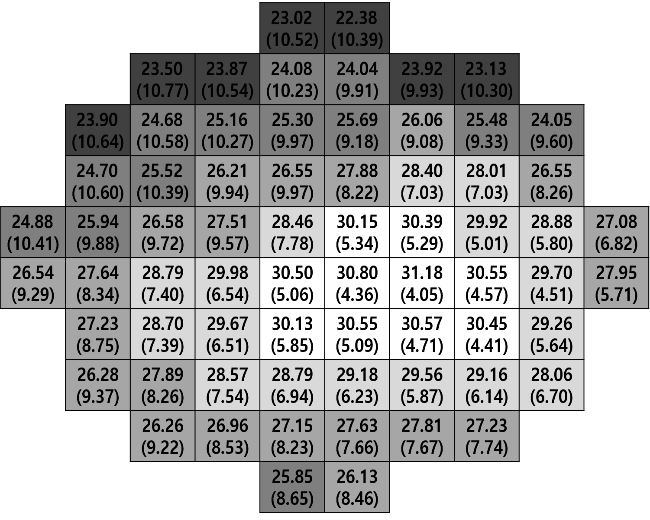

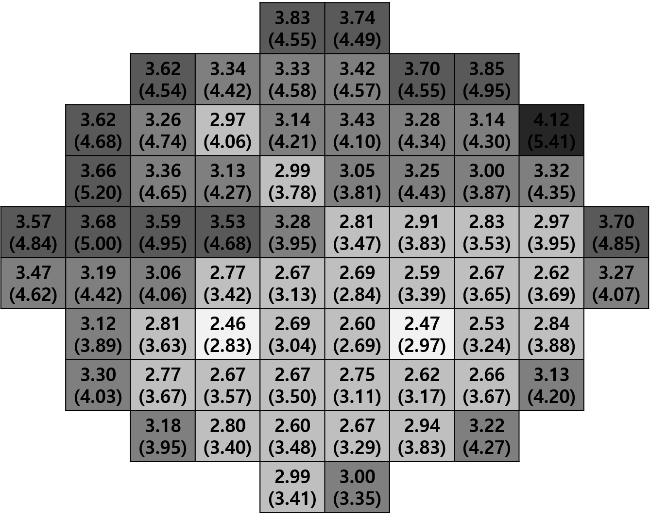


**c. RNFLT model d. *P*-values between the two models**

**
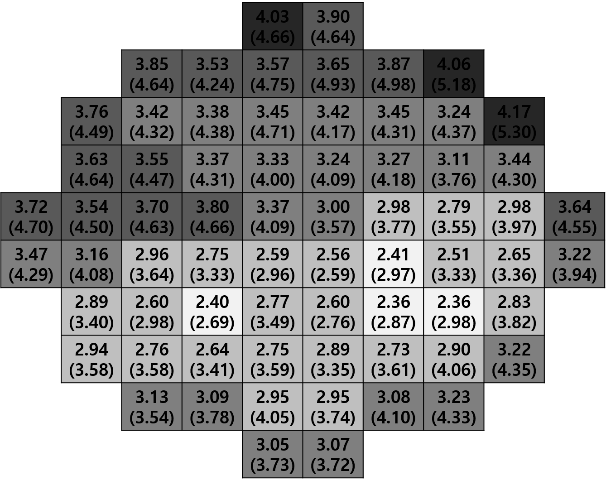

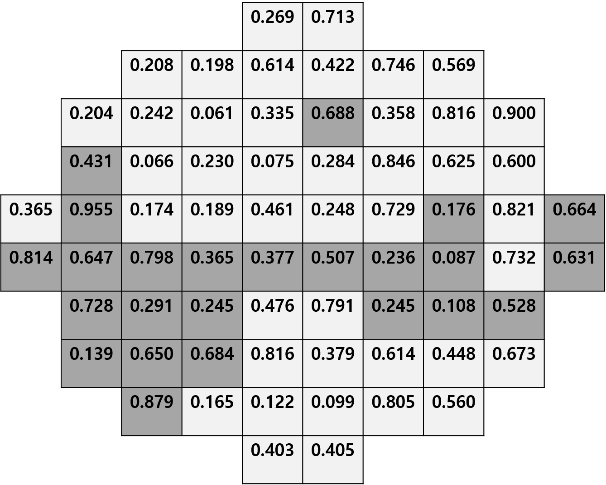
**

RNFLT = retinal nerve fiber layer thickness

Supplementary Fig. S4. Scatter plots of the prediction error versus the visual field mean deviation (MD). The slopes were −0.249 (R^2^ = 0.418, *P* < 0.001) in the en face model and −0.240 (R^2^ = 0.397, *P* < 0.001) in the RNFLT model, which suggested that as the MD decreased, the prediction error became greater.

**a. En face model b. RNFLT model**


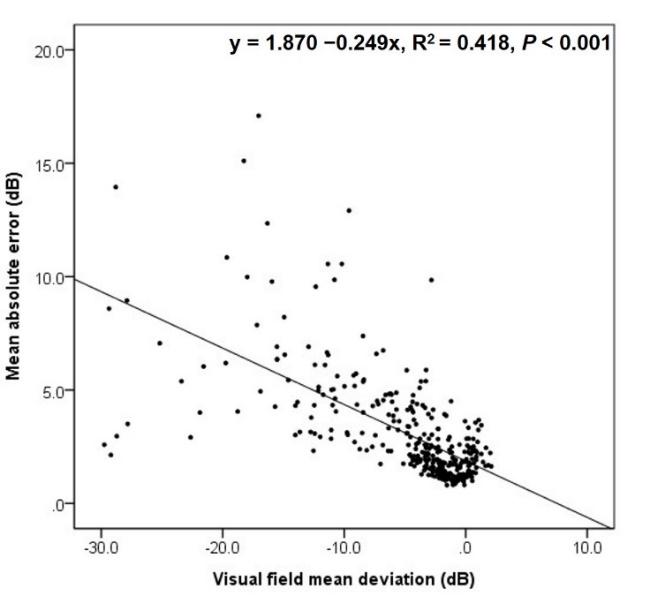

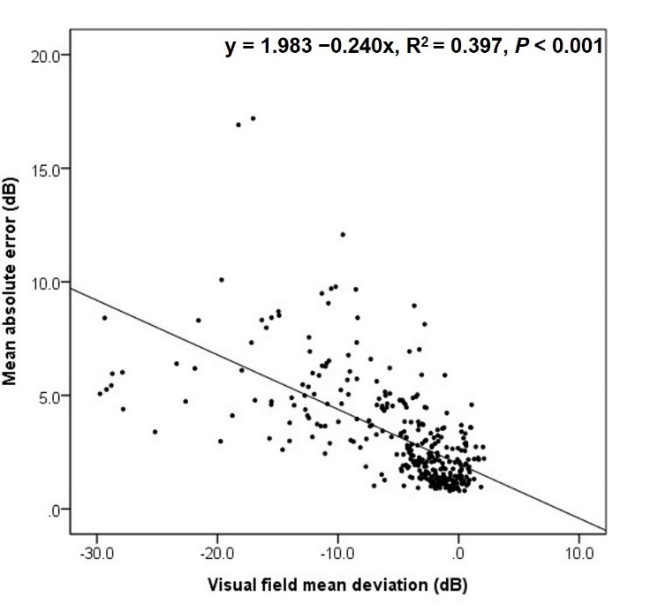


RNFLT = retinal nerve fiber layer thickness

Supplementary Table S1. Correlation coefficients and simple linear regression analyses between visual field prediction error and various factors.

|  | Correlation coefficients | | Simple linear regression analysis | | |
| --- | --- | --- | --- | --- | --- |
|  | Spearman’s rho | *P*-value | Slope | R^2^ | *P*-value |
| En face model | | | | | |
| Age | 0.101 | 0.065 | 0.017 | 0.008 | 0.094 |
| Sex | −0.097 | 0.076 | −0.570 | 0.014 | 0.031 |
| Visual acuity (logMAR) | 0.130 | 0.017 | 0.851 | 0.009 | 0.087 |
| Spherical equivalent | −0.147 | 0.007 | −0.083 | 0.008 | 0.104 |
| CCT | −0.141 | 0.016 | −0.008 | 0.016 | 0.031 |
| Axial length | −0.023 | 0.699 | −0.078 | 0.002 | 0.432 |
| 10-2 VF MD | −0.684 | <0.001 | −0.249 | 0.418 | <0.001 |
| OCT image quality | −0.132 | 0.015 | −0.019 | 0.003 | 0.307 |
| Average mGC/IPLT | −0.505 | <0.001 | −0.110 | 0.186 | <0.001 |
| Average cpRNFLT | −0.532 | <0.001 | −0.052 | 0.222 | <0.001 |
| RNFLT model | | | | | |
| Age | 0.142 | 0.009 | 0.021 | 0.013 | 0.039 |
| Sex | −0.106 | 0.052 | −0.453 | 0.009 | 0.083 |
| Visual acuity (logMAR) | 0.180 | 0.001 | 1.241 | 0.019 | 0.011 |
| Spherical equivalent | −0.125 | 0.022 | −0.062 | 0.005 | 0.219 |
| CCT | −0.108 | 0.065 | −0.007 | 0.015 | 0.039 |
| Axial length | −0.025 | 0.680 | −0.084 | 0.003 | 0.391 |
| 10-2 MD | −0.681 | <0.001 | −0.240 | 0.397 | <0.001 |
| OCT Image quality | −0.139 | 0.010 | −0.043 | 0.016 | 0.022 |
| Average mGC/IPLT | −0.598 | <0.001 | −0.132 | 0.277 | <0.001 |
| Average cpRNFLT | −0.586 | <0.001 | −0.056 | 0.267 | <0.001 |

logMAR = logarithm of the minimum angle of resolution; CCT = central cornea thickness; VF = visual field; MD = mean deviation; OCT = optical coherence tomography; mGC/IPLT = macular ganglion cell/inner plexiform layer thickness; cpRNFLT = circumpapillary retinal nerve fiber layer thickness

Supplementary Table S2. Multiple linear regression analyses between visual field prediction error and various factors

|  | Adjusted β | *P*-value | VIF |
| --- | --- | --- | --- |
| En face model |  |  |  |
| Age | −0.044 | 0.465 | 1.689 |
| Visual acuity (logMAR) | −0.090 | 0.082 | 1.273 |
| Spherical equivalence | −0.071 | 0.318 | 2.398 |
| CCT | −0.042 | 0.369 | 1.039 |
| Axial length | −0.137 | 0.067 | 2.631 |
| 10-2 VF MD | −0.701 | <0.001 | 2.162 |
| OCT Image quality | 0.013 | 0.792 | 1.234 |
| Average mGC/IPLT | 0.032 | 0.756 | 4.975 |
| Average cpRNFLT | −0.036 | 0.737 | 5.324 |
| RNLFT model |  |  |  |
| Age | −0.068 | 0.268 | 1.689 |
| Visual acuity (logMAR) | −0.081 | 0.133 | 1.273 |
| Spherical equivalence | 0.005 | 0.943 | 2.398 |
| CCT | −0.042 | 0.383 | 1.039 |
| Axial length | −0.100 | 0.194 | 2.631 |
| 10-2 MD | −0.588 | <0.001 | 2.162 |
| OCT image quality | −0.037 | 0.477 | 1.234 |
| Average mGC/IPLT | −0.210 | 0.048 | 4.975 |
| Average cpRNFLT | 0.083 | 0.449 | 5.324 |

Final models: Outcome = mean absolute error (MAE) of prediction. En face model; adjusted R^2^ = 0.456, *P* < 0.001, RNLFT model; adjusted R^2^ = 0.419, *P* < 0.001, Enter method used. No multicollinearity was found between variables (all VIFs ≤ 5.324). VIF = variance inflation factors; logMAR = logarithm of the minimum angle of resolution; CCT = central corneal thickness; VF = visual field; MD = mean deviation; OCT = optical coherence tomography; mGC/IPLT = macular ganglion cell/inner plexiform layer thickness; cpRNFLT = circumpapillary retinal nerve fiber layer thickness
